# IRF6 Is Directly Regulated by ZEB1 and ELF3, and Predicts a Favorable Prognosis in Gastric Cancer

**DOI:** 10.3389/fonc.2019.00220

**Published:** 2019-04-04

**Authors:** Dandan Li, Ping Cheng, Jingjie Wang, Xuemei Qiu, Xudong Zhang, Li Xu, Ying Liu, Shanshan Qin

**Affiliations:** ^1^Institute of Basic Medical Sciences, School of Basic Medical Sciences, Hubei University of Medicine, Shiyan, China; ^2^School of Biomedical Engineering, Hubei University of Medicine, Shiyan, China; ^3^Shiyan Hospital of Traditional Chinese Medicine, Shiyan, China; ^4^Hubei Key Laboratory of Wudang Local Chinese Medicine Research, Shiyan, China

**Keywords:** IRF6, ELF3, ZEB1, transcriptional regulation, gastric cancer

## Abstract

Interferon regulatory factor 6 (IRF6) acts as a tumor suppressor and controls cell differentiation in ectodermal and craniofacial tissues by regulating expression of target genes. However, its function in gastric cancer (GC) remains unknown to date. In this study, we found that the IRF6 expression was significantly downregulated in GC. And the decreased expression of IRF6 was clinically correlated with poor prognosis of GC. Moreover, loss-of-function and gain-of-function studies showed that IRF6 was negatively regulated by ZEB1 but positively regulated by ELF3. Additionally, transcription factor ZEB1 and ELF3 could directly bind on IRF6 promoter, which suggested that transcription factor IRF6 is transcriptionally regulated by ZEB1 and ELF3. Nevertheless, we found that IRF6 expression was negatively related to its promoter methylation in TCGA stomach cancer cohorts. The downregulation of IRF6 in GC might be due to the overexpression of ZEB1 and the DNA methylation of IRF6 promoter.

## Introduction

Gastric cancer (GC) is the third leading cause of cancer-related death worldwide, especially in East Asia (1). Despite a decrease in its incidence in some regions of the world, the 5-years overall survival rate remains unsatisfactory because most of GC patients are diagnosed in an advanced stage, with a poor prognosis and limited treatment options (2, 3). Although there is a great advancement on the gastric carcinogenesis, the molecular mechanisms underlying GC progression remains unclear (4). Hence, better understanding of the GC progression is essential to identify new effective diagnostic markers and novel effective therapies for GC patients.

Interferon regulatory factor 6 (IRF6) belongs to a family of nine transcription factors that share a highly conserved helix–turn–helix DNA-binding domain and a less conserved protein-binding domain (5). Unlike other IRF family members, IRF6 is not involved in IFN gene regulation, but instead have an essential role in skin development and keratinocyte differentiation (6, 7). Recent studies have proved that IRF6, regulated by TP63, plays a tumor suppressor role in squamous cell carcinomas through a Notch-dependent mechanism (8–10). However, the IRF6 gene expression regulation in gastrointestinal cancer types are not yet reported.

Previous studies have reported that IRF6 was downregulated during EMT process of breast cancer and prostate cancer (11–13). ZEB1 and ELF3 (E74 like ETS transcription factor 3) are two of transcription factors that involved in epithelial-mesenchymal transition (EMT) process. Transcription factor ZEB1 is known to be a master regulator of EMT process in varied cancer types (14, 15). Unlike the promotion of EMT by ZEB1, ELF3 is proved to be a negative regulator of EMT in ovarian cancer cells (16). Besides, it's recently reported that ELF3 functions as an antagonist of oncogenic-signaling-induced expression of EMT-TF ZEB1 in colorectal cancer (17). These results suggested that ELF3 and ZEB1 might play opposite roles in the EMT process of tumor cells.

In this study, we identified that the expression level of transcription factor IRF6 protein and mRNA was significantly downregulated in GC. Besides, the lower expression of IRF6 predicts poorer prognosis of GC. In addition, our data highlights that ZEB1 and ELF3 are two transcriptional regulators of IRF6 in GC; IRF6 is negatively regulated by ZEB1 but positively regulated by ELF3 in GC.

## Materials and Methods

### Expression Data Analysis

Six public gastric cancer microarray gene profiling datasets (GSE26942, GSE35809, GSE54129, GSE62254, GSE63089, and GSE79973) were downloaded from the Gene Expression Omnibus (GEO) in the NCBI web server. The clinical information of GSE62254 gastric cancer cohort was download from Cristescu et al. (18). The expression data of normal stomach tissue and stomach cancer tissues was obtained from GTEx and TGCA, respectively by using GEPIA (http://gepia.cancer-pku.cn/index.html) and UCSC (http://genome.ucsc.edu/). The chip-seq data of ELF3 and ZEB1 download from NCBI was analyzed by Cistrome (http://cistrome.org/db/#/). The chip-seq data of Dnase I, H3K27Ac, EP300, SRF, POLR2A, MED1, CREB1 and BRD4 were obtained from http://cistrome.org/browser/?genome=hg38wugb&datahub=http://dc2.cistrome.org/data5/browser/1535255763.json&gftk=refGene,full&coordinate = chr7:27066839-27266927. The possible binding sites of ELF3 and ZEB1 in the 2000-length IRF6 promoter was predicted by JASPAR (http://jaspar.genereg.net/). The correlation of IRF6 promoter methylation and IRF6 expression was analyzed by MEXPRESS (https://mexpress.be/) and LinkedOmics (http://www.linkedomics.org/login.php).

### Cell Culture

The human gastric cancer cell line (N87, BGC823, AGS, SGC7901, MGC803, and HGC27) and a normal gastric epithelium cell line (GES-1) were purchased from the Shanghai Cell Bank of Chinese Academy of Sciences (Shanghai, China). All the GC cell lines and the GES-1 Cell line were cultured in DMEM medium containing 10% fetal bovine serum (FBS), 100 U/mL penicillin,100 U/mL streptomycin and 0.03% glutamine at 37°C in 5% CO_2_.

### Cell Transfection

The siRNAs (si-ELF3#1: 5′-GCUGCAACCUGUGAGAUUA-3′, si-ELF3#2: 5′-CC-UCUGCAAUUGUGCCCUU-3′, si-ELF3#3: 5′-CCAUGAGGUACUACUACAA-3′; si-ZEB1: 5′-UGAUCAGCCUCAAUCUGCA-3′, si-NC: 5′-UUCUCCGAACGUGUCACG U-3′) used to knock down ELF3 and ZEB1 expression in GC cell lines were designed and synthesized by Genepharma (Shanghai, China). The ORF region of ZEB1 cDNA was cloned into pcDNA3.1 (+). Gastric cancer cells were grown in 6-well plates and transfected by Lipofectamine 2000 (Invitrogen) according to the manufacturer's instructions. At 48 h post-transfection, cells were harvested for qPCR analysis. For ELF3 overexpression experiment, ELF3 and controlled scrambled plasmids were purchased from GeneChem (Shanghai, China). Transfection was performed with Lipofectamine 2000 (Invitrogen) according to the manufacturer's instructions.

### RNA Extraction and Quantitative RT-PCR

For RNA extraction, gastric cancer cells were grown in 6-well plates and transfected with siRNAs. After 48 h, remove the medium and directly add 800 μL Trizol into the 6-well plate to harvest samples. Total RNA was extracted using Trizol reagent (Invitrogen, USA) according to the manufacturer's instructions. The isolated RNA was treated with RNase-free DNase I (Roche) for 15–30 min as we described before (19). PCR was performed to ensure removal of genomic DNA by using RNA samples as templates. Reverse transcription was performed to obtain cDNA using the PrimeScript™ RT reagent Kit (Perfect Real Time, Takara) according to the manufacturer's instructions. For Quantitative RT-PCR, all the cDNA samples were diluted 5 times. The qPCR protocol was using One Step TB Green PrimeScript™ RT-PCR Kit II (Takara) according to the manufacturer's instructions. The qPCR analysis was conducted on Bio-Rad CFX Manager 3.1 real-time PCR system. The Cycling conditions were as follows: 95°C for 30 s, 95°C for 5 s and 60°C for 30 s. The reaction was performed for 40 cycles. The mRNA expression of ELF3, IRF6, and ZEB1 were determined by using the specific primers (ELF3-F: 5′-CACTGATGGCAAGCTCTTC-3′, ELF3-R: 5′-GGAGCG-CAGGAACTTGAAG-3′; IRF6-F: 5′-CCAGTAGTGGCTCGGATGAT-3′, IRF6-R: 5′-CAGCTCTCCTGGGTTTG AAG-3′; ZEB1-F: 5′-ACCTCTTCACAGGTTGCTCCT-3′, ZEB1-R: 5′-AGTGC AGGAGCTGAGAGTCA-3′, ACTIN-F: 5′-ATCGTCCACCGCA-AATGCTTCTA-3′, ACTIN-R: 5′-AGCCATGCCAATCTCATCTTGTT-3′). The experiment was performed with three replicates and average values were presented. Comparative quantification was determined using the 2^−ΔΔCt^ Method.

### Dual Luciferase Reporter Assay

For IRF6 promoter cloning, total genomic DNAs were extracted from AGS cell lines using TIANamp Genomic DNA Kit (TIANGEN, China). The 1,000 bp-length of IRF6 promoters were amplified by PCR (forward primer: GTGACCCATGCCTATATTT, reverse primer: GGGCGCCTGGCTCTACCCAA) and then clone into pMD18-T vectors (Takara) which were next used as templates for wildtype and mutant IRF6 promoters cloning. For reporter vectors construction (WT), the wildtype promoters of IRF6 (NM_006147) were amplified by PCR (forward primer: actagtacgcgtatttGAAAGAGAAAAAAGCAAACA, reverse primer: agagtttaaacgtcgacatttGGGCGCCTGGCTCTACCCAA). After digesting the pEZX-FR01 vector (GeneCopoeia, USA) into a linear fragment by SwaI, the IRF6 promoters and the linear vector fragments are recombined by seamless cloning (Yeasen Biotech, China) according to the manufacturer's instructions. For reporter vectors construction (Mut), two DNA fragments (mutant IRF6 promoter) was amplified by using the templates that mentioned above. The primers are as follows: F1, actagtacgcgtatttTTGAACTGGGTGCCA; R1, GATCGATCTTCTTTTTTGTTGTTG; F2, AAAAGAAGATCGATCTTATTATTCTCATTG, R2, agagtttaaacgtcgacatttTCTCCCCGTCCCGCAC. After digesting the pEZX-FR01 vector into a linear fragment by SwaI, the linear vector fragments and the mutant IRF6 promoters are recombined by seamless cloning according to the manufacturer's instructions. AGS and SGC7901 cells were seeded into 12-well-tissue plates 24 h before transfection, and then co-transfected with 5 ng siRNA and 1 mg plasmid using the Lipofectamine 2000 Reagent (Invitrogen), according to the manufacturer's instructions. After another 48 h, cells were assayed using the Dual-Luciferase reporter assay system kit (GeneCopoeia, USA). All experiments were performed in triplicate and data were pooled from three independent experiments.

### Chromatin Immunoprecipitation Assay

Chromatin immunoprecipitation (CHIP) assays were performed using CHIP Assay Kit (56383S, Cell Signal Technology, USA) according to the manufacturer's protocol. ELF3 antibody were purchase from Santa Cruz Biotechnology (sc-376055, USA); ZEB1 antibody were purchase from Santa Cruz Biotechnology (sc-25388, USA). Briefly, SGC7901 cells were collected and fixed for 10 min at 37°C with 1% formaldehyde, followed in sequence with SDS lysis and DNA shearing, protein and DNA immunoprecipitation, cross-linked DNA reversal and DNA purification, and finally the immunoprecipitated DNA fragments were detected by PCR assays. The normal rabbit IgG was used as the negative control. The sequence of primers for CHIP (anti-ELF3) were used as follows (forward: TCTGAACTCCCAGTCGCTTC; reverse: TATGACACTCCG CGTTTCTG); the sequence of primers for CHIP (anti-ZEB1) were used as follows (forward: CGGGCGGATGCGAAGGCT; reverse: GGGCGCCTGGCTCTACCCAA).

### Statistical Analysis

Data from at least three independent experiments performed in triplicate were presented as the mean ± S.D. The differences between two groups were determined by student's *t*-test. Differences among multiple groups were determined by one-way ANOVA. Comparisons were performed using the Spearman's correlation test; *p* < 0.05 was considered statistically significant.

## Results

### Reduced IRF6 Expression Was Clinically Correlated With Poor Prognosis in GC

**Figure 1:**
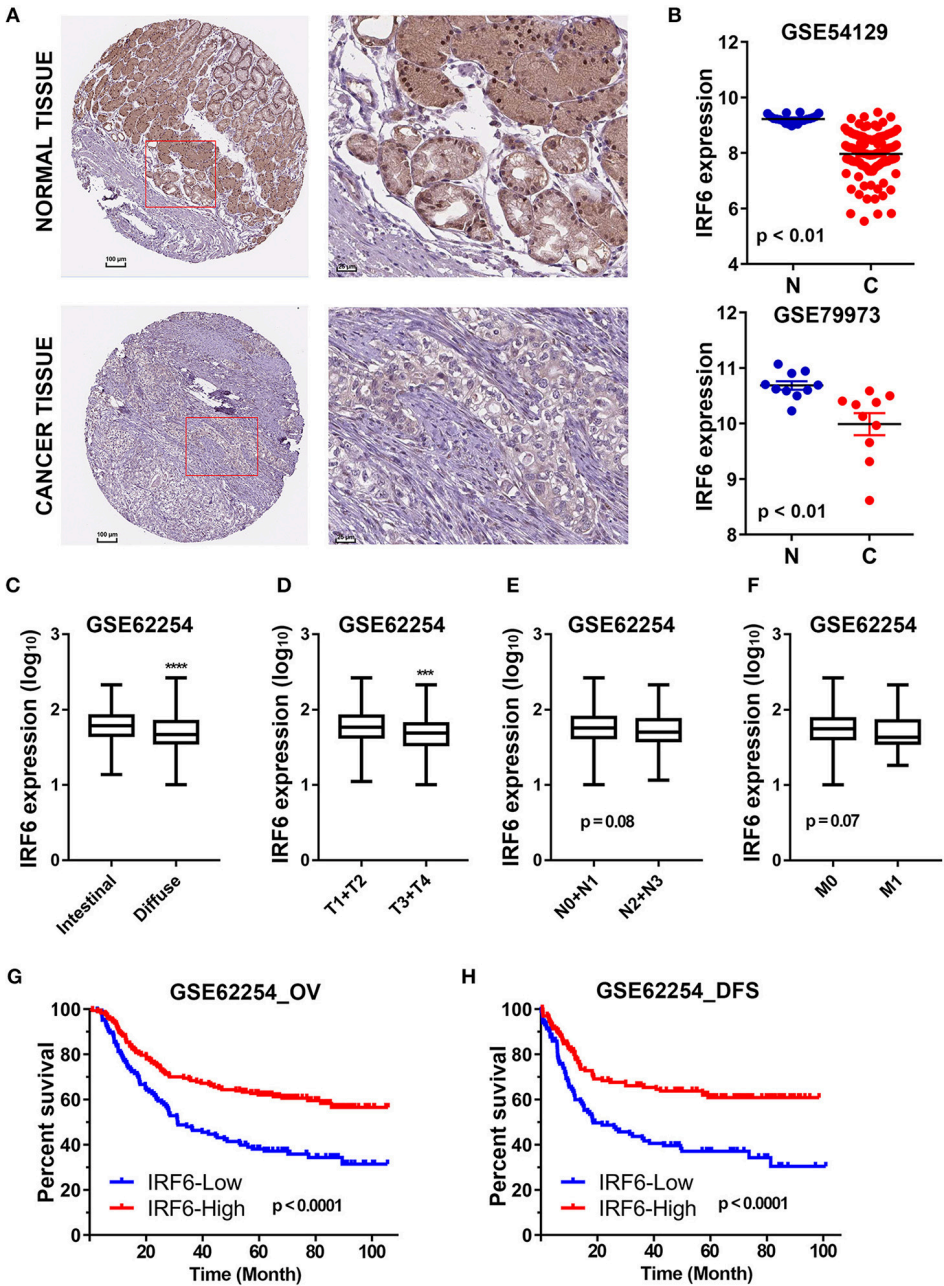
The decreased IRF6 expression predicted poor prognosis of gastric cancer. **(A)** The immunohistochemistry images of IRF6 in gastric cancer tissues and normal stomach tissues that obtained from HPA datasets. **(B)** Analysis of IRF6 expression data in GSE54129 and GSE79973. **(C)** The IRF6 expression level in diffuse and intestinal types of GC. **(D–F)** The IRF6 expression level in different T-stages **(D)**, N-stages **(E)**, and M-stages **(F)** of GC. **(G,H)** Gastric cancer patients with lower expression of IRF6 predicts possess shorter overall survival time **(G)** and disease free time **(H)**. ^****^p < 0.0001, ^***^p < 0.001.

The Human Protein Atlas (HPA) is an effort to map the subcellular location of all human proteins (http://www.proteinatlas.org/), which contains a large number of immunohistochemistry (IHC) images of sections from 46 different normal human tissue types and 20 different cancer types (20). To evaluate subcellular antibody staining patterns of IRF6 in normal and cancer tissue of stomach, the corresponding IHC images of IRF6 were downloaded from HPA web server. The results showed that IRF6 proteins were mainly located in cytoplasm and nucleus of glandular cells in normal stomach tissues and rarely expressed in cancerous tissues (Figure 1A). Furthermore, we analyzed the expression of IRF6 in two microarray gene profiling data (GSE54129 and GSE79973) that contained expression data of both normal tissues and cancer tissues. The results showed that the transcripts level of IRF6 in the gastric cancer tissues was lower than in the corresponding normal tissues (Figure 1B).

In order to understand the significance of decreased IRF6 expression in gastric cancer, we further determined the potential associations between IRF6 expression level and clinicopathological features in GSE62254 GC cohorts (*n* = 300). The results showed that diffuse GC tissues possessed lower IRF6 expression than intestinal GC tissues (Figure 1C). Moreover, IRF6 expression level was negatively correlated with advanced clinical TNM stages (Figures 1D–F). In addition, GC patients with higher expression of IRF6 tended to possess longer overall survival time and disease free time than patients with lower expression of IRF6 (Figures 1G,H). These results together suggested that lower IRF6 expression predicted poorer prognosis in GC.

### IRF6 Was Directly Regulated by ZEB1 in GC

**Figure 2:**
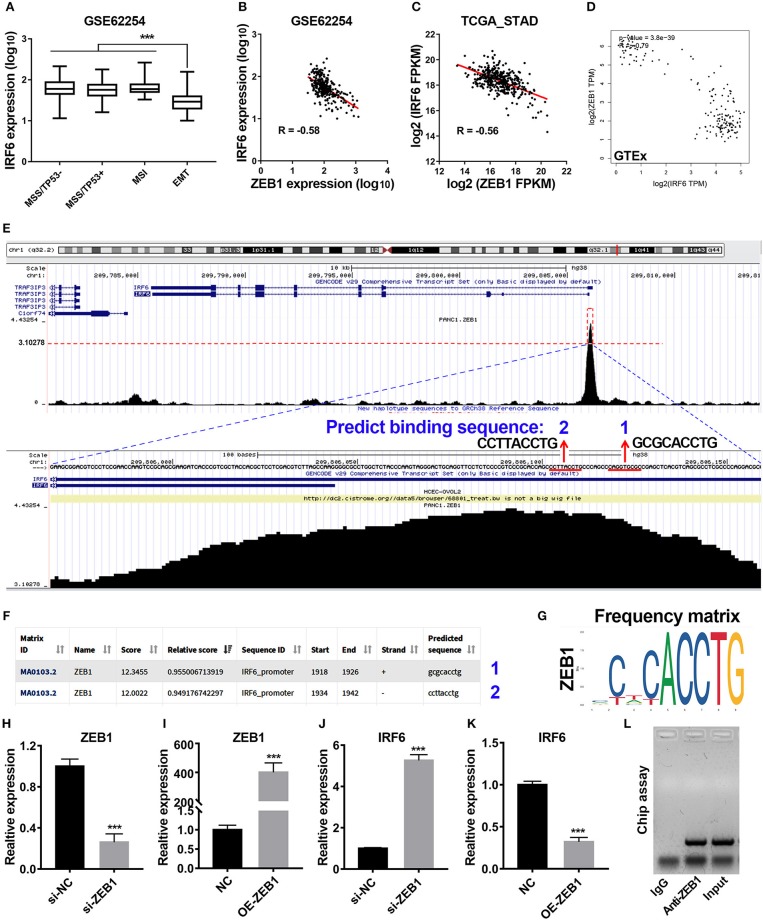
Transcription factor ZEB1 negatively regulates IRF6 expression. **(A)** The expression level of IRF6 in diffuse and intestinal gastric cancer. **(B–D)** The expression correlation of ZEB1 and IRF6 in GSE62254 gastric cancer cohort **(B)**, TCGA gastric cancer cohort **(C)**, and normal stomach tissues cohort in GTEx **(D)**. **(E)** The chip-seq data of ZEB1 is analyzed by using UCSC web tool. **(F)** The 2,000 bp-length of IRF6 promoter was analyzed by JASPAR web tool to predicted possible ZEB1 binding sites. **(G)** The frequency matrix of ZEB1 binding sequence was obtained from JASPAR. **(H–K)** Knockdown **(H)** or overexpression **(I)** of ZEB1 results in a significant increase of IRF6 expression **(J)** or a remarkable decrease of IRF6 expression **(K)** in GC, respectively. **(L)** CHIP assay showing the binding of ZEB1 to IRF6 promoter *in vivo*. The ZEB1 protein was pulled down in SGC7901 cells, and specific primers were used to amplify the IRF6 promoter in the recovered DNA from the IP complex. Non-specific IgG are used as controls. ^***^p < 0.001.

Previous study has reported that gastric cancer should be divided into four subtypes, including MSI, MSS/TP53+, MSS/TP53–, and MSS/EMT (18). During analysis of correlation between IRF6 expression level and clinicopathological features in GSE62254 GC cohorts, we noted that IRF6 expression in MSS/EMT subtype was remarkably lower than other three subtypes (Figure 2A). As ZEB1 was a master regulator of EMT process in many cancer types, we firstly considered to analyze the expression correlation of ZEB1 and IRF6 in GC. The results showed that IRF6 expression level was negatively associated with ZEB1 expression in both GC tissues and normal stomach tissues (Figures 2B–D).

To identify if ZEB1 could directly regulate IRF6 expression in GC, we firstly analyzed the available chip-seq data of ZEB1 using the online website of Cistrome. The result showed that an obvious ZEB1 peak was observed in the promoter region of IRF6 in different cell lines (Figure S1). Here, we took the chip-seq of ZEB1 in pancreatic cancer cell line as an example for further analysis. As shown in the bottom of Figure 2E, the most abundant section in the IRF6 promoter region pulled down by the ZEB1 antibody was located in 110 bp upstream of TSS of IRF6 (NM_006147). Besides, this section contained two ZEB1 binding sites predicted by JASPAR web server (Figures 2E–G), which suggested that IRF6 might be transcriptionally regulated by ZEB1. In order to confirm this possibility, we examined the IRF6 expression level after knockdown and overexpression of ZEB1 in AGS cell line (Figures 2H,I). The results showed that knockdown expression of ZEB1 significantly increased IRF6 expression; while overexpression of ZEB1 significantly decreased IRF6 expression (Figures 2J,K). Additionally, the result of CHIP assay showed that ZEB1 could directly bind on the IRF6 promoters in gastric cancer cells (Figure 2L). These results together indicated that transcription factor ZEB1 negatively regulated IRF6 expression via directly binding on IRF6 promoter.

### IRF6 Was Positively Regulated by ELF3 in GC

**Figure 3:**
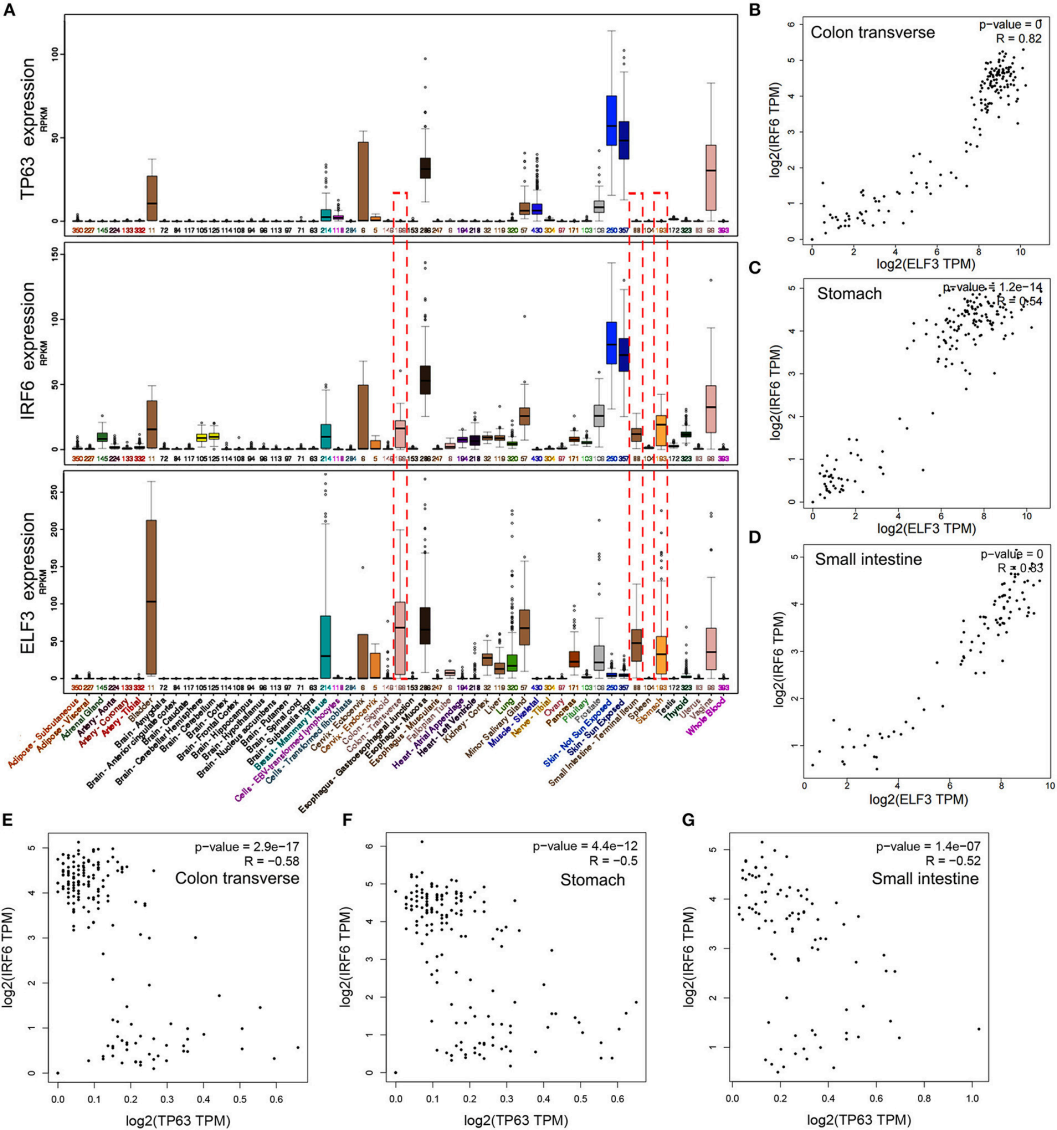
The expression profile of IRF6 and ELF3 was similar in most of normal human tissues. **(A)** The expression profile of IRF6, ELF3, and TP63 in varies kinds of normal human tissues according to GTEx. **(B–D)** The expression correlation of ELF3 and IRF6 in the normal human colon **(B)**, stomach **(C)**, and small intestine **(D)** according to GTEx. **(E–G)** The expression correlation of TP63 and IRF6 in the normal human colon **(E)**, stomach **(F)**, and small intestine **(G)** according to GTEx.

IRF6 was reported to be positively regulated by TP63 in squamous cell carcinomas and normal skin tissues. To verify if the regulation of TP63 on IRF6 expression exists in other tissues, we downloaded the expression data of IRF6 and TP63 in varies kinds of normal tissues from GTEx Portal. Based on comparative analysis, we found that TP63 and IRF6 showed a very similar expression profile in most kinds of tissues, especially in human skin tissue. However, a certain degree of IRF6 expression can still be detected in the normal human tissues that rarely express TP63, such as stomach, colon and small intestine (Figure 3A). Therefore, there must be other genes that can regulate IRF6 expression in the human gastrointestinal tissues.

Transcription factors tends to possess similar expression patterns to their target genes. To identify which gene regulated IRF6 expression in human gastrointestinal tissues, we analyzed expression of all the transcription factors that co-expressed with IRF6. And the result indicated that transcription factor ELF3 showed a very similar expression profile with IRF6 in most kinds of tissues (Figure 3A). Furthermore, by comparing the correlation of between ELF3 and IRF6 expression in stomach, colon and small intestine tissues, we found that the IRF6 expression was highly relevant to ELF3 in the normal human gastrointestinal tissues (Figures 3B–D). Moreover, no obvious expression correlation was observed between TP63 and IRF6 in the normal human gastrointestinal tissues (Figures 3E–G).

**Figure 4:**
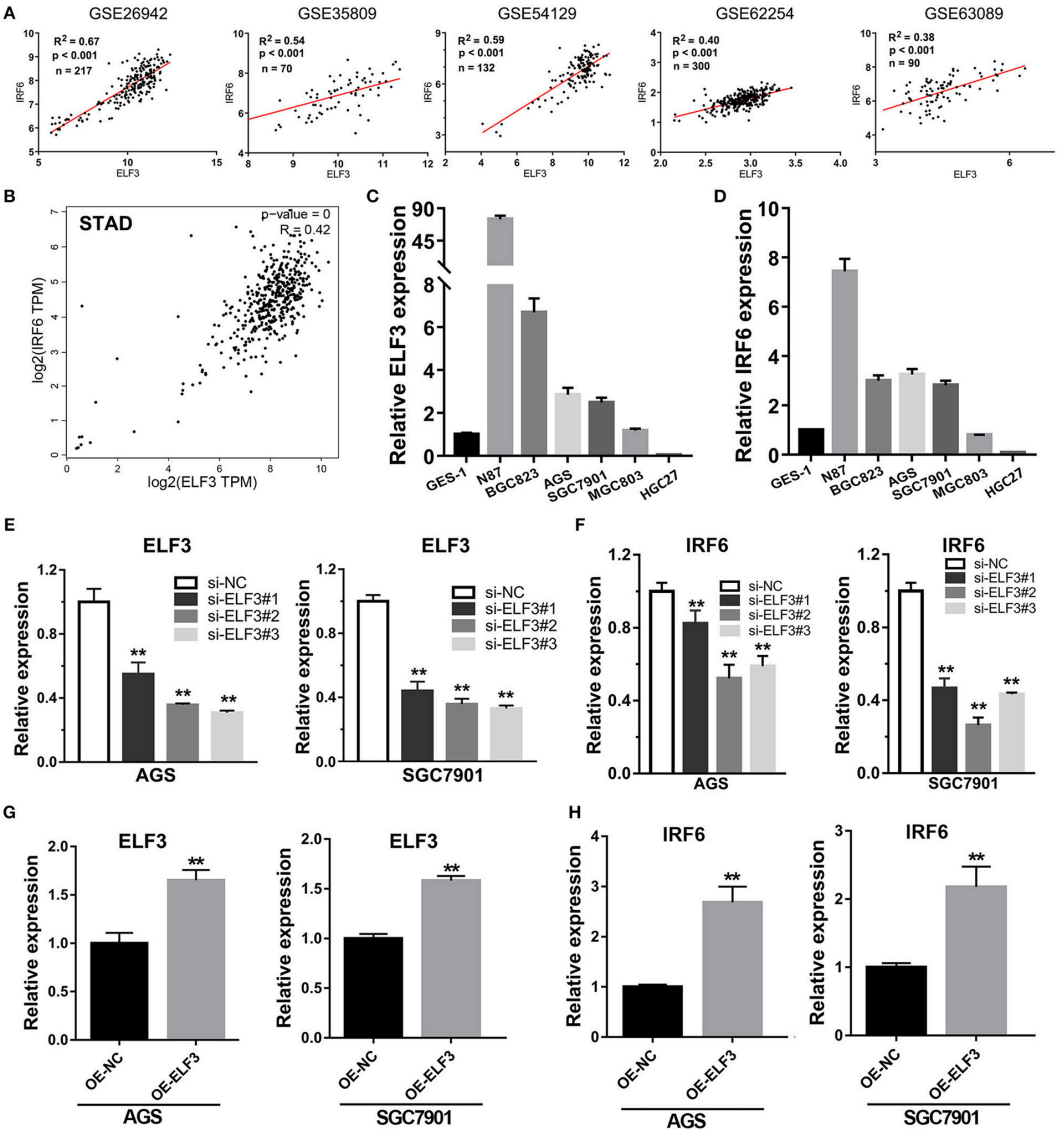
Transcription factor ELF3 positively regulates IRF6 expression in GC. **(A)** The expression correlation of ELF3 and IRF6 in five public gastric cancer microarray gene profiling datasets. **(B)** The expression correlation of ELF3 and IRF6 in gastric cancer tissues according to TCGA. **(C,D)** The expression level of ELF3 **(C)** and IRF6 **(D)** in six gastric cancer cell lines and the normal gastric epithelium cell line (GES-1). **(E)** The RNA inference efficiency of siRNAs that targeted to ELF3 in GC cell lines. **(F)** Knockdown of ELF3 declined IRF6 expression level in GC cell lines. **(G)** The ELF3 overexpression efficiency was determined by qPCR in GC cell lines. **(H)** Overexpression of ELF3 increased IRF6 expression level in both two GC cell lines. ^**^p < 0.01.

Considering highly similar expression profile between ELF3 and IRF6 in normal human stomach tissues, we wanted to know if ELF3 and IRF6 were still co-expressed in the gastric cancer tissues. To explore the ELF3 and IRF6 expression profile in human GC tissues, five microarray gene profiling data were downloaded from GEO datasets. The GSE26942 dataset consists of 217 GC samples; GSE35809 consists of 70 GC samples; GSE54129 consists of 132 GC samples; GSE62254 consists of 300 GC samples; GSE63089 consists of 45 paired samples. It was identified that ELF3 was co-expressed with IRF6 in all five gastric cancer GSE datasets (Figure 4A). Furthermore, we investigated and analyzed the expression of ELF3 and IRF6 in GC tissues from TCGA by using the bioinformatics tool GEPIA, a web server for cancer and normal gene expression profiling and interactive analyses (21). The results showed that ELF3 and IRF6 were highly co-expressed in GC tissues form TCGA (Figure 4B). In addition, after examination of ELF3 and IRF6 expression in six GC cell lines and the normal gastric epithelial cells GES-1 by performing qRT-PCR analysis, we found that ELF3 and IRF6 were both overexpressed in N87, BGC823, SGC7901 and AGS, but both hardly expressed in HGC27 compared to their expression level in GES-1 (Figures 4C,D). These results together indicated that the expression of ELF3 and IRF6 in GC tissues and cell lines were highly relevant.

However, it is still unclear whether ELF3 could regulate IRF6 or not. Thus, loss-of-function and gain-of-function studies were conducted in GC cell lines. Here, we chose SGC7901 and AGS gastric cancer cell lines to perform the RNA interference experiment and ELF3 overexpression experiment. The results showed that knockdown of ELF3 expression in GC cell lines significantly decreased IRF6 expression (Figures 4E,F), while overexpression of ELF3 in GC cell lines remarkably increased IRF6 expression (Figures 4G,H). These results suggested that IRF6 was positively regulated by transcription factor ELF3 in GC.

### ELF3 Directly Binds on the Promoter of IRF6

To evaluate if ELF3 directly regulate IRF6 expression or not, we firstly analyzed the only available chip-seq data of ELF3 antibody in the pancreatic ductal carcinoma, which obtained from the online website of Cistrome (22). As expected, an obvious ELF3 peak was observed in the 700 bp-length of IRF6 promoter (Figure S2A). Coincidentally, bioinformatics analysis shows that the 2,000 bp-length promoter contained a possible ELF3 binding site, which was located at 660–672 bp upstream of IRF6 transcription start site (Figure S2B).

**Figure 5:**
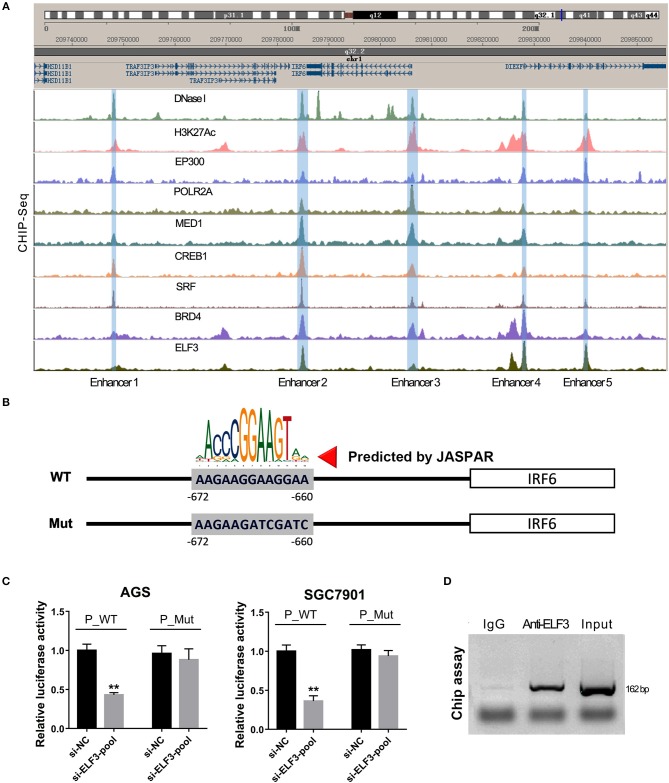
ELF3 directly bound on the promoter of IRF6 in gastric cancer. **(A)** The Cistrome browser diagram showed that five enhancers were located near the IRF6 gene; ELF3 could bind on the region of all enhancers near the IRF6 gene. **(B)** The 700 bp length of IRF6 promoter contained six putative ELF3 binding sites. **(C)** GC cell lines (AGS and SGC7901) were co-transfected with siRNA pool targeted to ELF3 and luciferase reporter vector. After 48 h of incubation, luciferase activity was measured. **(D)** CHIP assay showing the binding of ELF3 to IRF6 promoter *in vivo*. The ELF3 protein was pulled down in SGC7901 cells, and specific primers were used to amplify the IRF6 promoter in the recovered DNA from the IP complex. Non-specific IgG are used as controls. ^**^p < 0.01.

Furthermore, our unexpected findings showed that ELF3 might regulate IRF6 expression via binding on the enhancers near the IRF6 gene. Recent genome-wide studies have established that enhancers can be defined as DNA sequences that bind the transcriptional co-activator p300/CREB1/SRF/MED1/BRD4, that bind H3K27AC, and then recruit RNA polymerase II to start transcription (23). Therefore, we analyze the available chip-seq data of these transcription factors according to the online website of Cistrome. The results showed that there are five enhancers located near the IRF6 gene (from 50 kb downstream of IRF6 gene to 50 kb upstream of IRF6 gene). An obvious ELF3 binding peak was found in all the five enhancer regions, which suggested that ELF3 might be a transcriptional regulator of IRF6 (Figure 5A). Moreover, it is worth mentioning that the enhancer 3 was located at 700 bp-length promoter of IRF6.

To detect the ELF3 binding activity on the IRF6 promoter, we designed a mutant IRF6 promoter that the predicted ELF3 binding sites by JASPAR (Figure 5B). After the two IRF6 promoter sequences were cloned into the luciferase reporter vector, the luciferase activity was examined in the AGS and SGC7901 cell lines that transfected with ELF3 siRNAs pool and negative control siRNA. As expected, a significant decrease was observed in the both AGS and SGC7901 cell that transfected with ELF3 siRNAs pool and wildtype IRF6 promoter luciferase reporter, compared to the cell transfected with negative control siRNA and wildtype IRF6 promoter luciferase reporter. However, no obvious changes were detected in the AGS and SGC7901 cell that transfected with mutant IRF6 promoter luciferase reporter (Figure 5C). In addition, chromatin immunoprecipitation (CHIP) result in SGC7901 further confirmed that ELF3 could indeed directly bind to the promoter of IRF6 (Figure 5D). These results together indicated that ELF3 was also a transcriptional regulator of IRF6.

## Discussion

Interferon regulatory factors (IRFs) family, consisting of nine members in mammals, commonly possessed a novel helix-turn-helix DNA-binding domain and was proved to be involved in regulation of immunity and oncogenesis (24). Transcription factor IRF6, as a member of IRF family, was reported to play an essential role in keratinocyte differentiation, craniofacial development, salivary Glands and pancreas development (7, 25, 26). While the function of IRF6 in cancers has only been reported in squamous cell carcinomas and breast cancer. In squamous cell carcinomas, IRF6 expression is downregulated and knockdown of IRF6 promotes invasive behavior of tumor cells. In breast cancer, IRF6 overexpression results in a significant reduction of breast cancer cell numbers through arresting cell cycles (27). In other words, IRF6 plays a tumor suppressor role in squamous cell carcinomas and breast cancer. However, in addition to high expression in the breast and skin tissues, IRF6 is also highly expressed in gastrointestinal tissues according to analysis of the RNA-seq data in GTEx dataset (Figure 3A). Up to now, the expression profile and function of IRF6 in gastrointestinal cancer has not been reported yet.

In this study, we determined to investigate the function of transcription factor IRF6 in gastric cancer. Firstly, we identified that the expression levels of IRF6 protein and transcript are both significantly reduced in GC compared with normal stomach tissues (Figures 1A,B). Besides, the decreased expression level of IRF6 was closely related to malignant progression and poor prognosis of gastric cancer (Figures 1C–H). These results strongly suggested that IRF6 might function as a tumor suppressor in GC, which was similar to the role of IRF6 in squamous cell carcinomas and breast cancer. Therefore, we speculated that the mechanism of IRF6 downregulation and anti-cancer effect might be conservative at least in these cancers. Due to the reason why IRF6 was downregulated in these 3 cancer types remains largely unclear, our following studies focused on identifying the possible transcriptional regulators of IRF6 in gastric cancer.

Previous studies have reported that IRF6 was downregulated during EMT process of breast cancer and prostate cancer (11–13). Our results also showed that IRF6 was lowly expressed in MSS/EMT subtype of GC. Besides, a significant negative correlation between expression of ZEB1 and IRF6 was observed in GC. And the CHIP assay in gastric cancer cells showed that ZEB1 could bind on IRF6 promoter. More importantly, loss-of-function and gain-of-function studies showed that IRF6 was negatively regulated by ZEB1. Taken together, we firstly confirmed that transcription factor ZEB1 was a negative transcriptional regulator of IRF6 in GC. It is worth noting that lots of evidences show that transcription factor ZEB1 was overexpressed in gastrointestinal cancers (28–30). Therefore, the downregulation of IRF6 in GC or in EMT process of GC, SCC and breast cancer may be due to upregulation of ZEB1.

In addition, previous studies showed that IRF6 is a downstream target gene of the NOTCH signaling pathway and induced by the NOTCH signaling pathway in breast cancer and keratinocytes (9, 31, 32). NOTCH signaling pathway has been reported to play critical roles in the development and progression of human cancers through regulating ZEB1 expression and EMT pathway (33–38). For instance, knockdown of NOTCH3 upregulated ZEB1 expression in esophageal squamous cell carcinoma (39). Interestingly, analysis of microarray data of NOTCH3 knockdown (GSE27424) showed that silencing NOTCH3 results in a significant increase of ZEB1 and a significant decrease of IRF6 in esophageal squamous cell carcinoma. Based on our current findings, it is highly probable that the NOTCH signaling pathway regulates IRF6 expression through regulation of ZEB1.

On the other hand, we tried to identify possible transcriptional regulators that positively regulate IRF6 expression. Due to IRF6 was known to be a target gene of TP63 in skin development and squamous cell carcinomas, we firstly attempted to analyze the co-expression of IRF6 and TP63 in human gastrointestinal tissues. However, we found that a certain degree of IRF6 expression can still be detected in the human gastrointestinal tissues that hardly express TP63, such as stomach, colon and small intestine (Figures 3A,E–G). Therefore, there must be other genes that can regulate the expression of IRF6 in the human gastrointestinal tissues.

Inspired of this, we considered to analyze all the transcription factors that highly co-expressed with IRF6 and finally identified ELF3 as a possible positive transcriptional regulator of IRF6 in GC due to ELF3 was highly co-expressed with IRF6 in normal stomach tissues, GC tissues and GC cell lines (Figures 3C, 4A–D). In addition, knockdown of ELF3 significantly decreased IRF6 expression and overexpression of ELF3 significantly increased IRF6 expression in GC cell lines (Figures 4E–H). Besides, it's identified that there are at least five enhancers located near the IRF6 gene and transcription factor ELF3 could bind on all the enhancers (Figure 5A). Additionally, the dual luciferase reporter and CHIP assay showed that ELF3 could directly bind on the promoter of IRF6 (Figures 5B–D). These results together indicate that ELF3 is a transcriptional regulator of IRF6 in GC.

Nevertheless, previous studies has reported that promoter DNA methylation at CpG islands is a common mechanism used by cancer cells to repress expression of tumor suppressor genes (40). Besides, it's reported that IRF6 is aberrantly silenced by DNA methylation of the 5′ IRF6 CGI in melanoma and squamous cell carcinomas (10, 41). Therefore, we also analyzed the correlation between IRF6 expression and promoter DNA methylation in GC tissues from TCGA by bioinformatics method. As expected, a significant negative correlation between IRF6 expression and promoter DNA methylation was observed, suggested promoter DNA methylation was also a potent reason of IRF6 downregulation in GC (Figure S3).

In conclusion, transcription factor IRF6 was downregulated in GC. The reduced expression of IRF6 predicted poor prognosis of GC. In addition, ZEB1 is identified to be a negative transcriptional factor of IRF6 gene, while ELF3 is identified to be a positive transcriptional factor of IRF6 gene in GC. The downregulation of IRF6 in GC might be owing to overexpression of ZEB1 and the DNA methylation of IRF6 promoter.

## Author Contributions

The research was designed by YL and SQ. The experiments were performed by SQ, DL, PC, JW, XQ, XZ, and LX, and the data were analyzed by SQ. The manuscript was written by SQ.

### Conflict of Interest Statement

The authors declare that the research was conducted in the absence of any commercial or financial relationships that could be construed as a potential conflict of interest.

## Supplementary Material

Figure S1 The chip-seq data of ZEB1 in different cancer cell lines was analyzed by Cistrome web server. An obvious peak located in the promoter region of IRF6 was observed in blood, kidney, pancreas, lung, and breast tissues.

Figure S2 Bioinformatics analysis showed that ELF3 might be a transcriptional regulator of IRF6. **(A)** The chip-seq data of ELF3 in pancreatic cancer cell lines was analyzed by Cistrome web server. An obvious peak located in the 700 bp-length of promoter region of IRF6 was observed **(B)** The possible ELF3 binding sites in 2,000 bp-length of IRF6 promoter was analyzed by JASPAR.

Figure S3 The IRF6 expression was negatively related to IRF6 promoter methylation. **(A)** An obvious CpG islands was located in the promoter region of IRF6. **(B)** The association between IRF6 expression and IRF6 promoter methylation in the TCGA stomach cancer cohort was analyzed by MEXPRESS web tool. **(C)** Genes that negatively correlated with IRF6 methylation was analyzed by using LinkedOmics web server. **(D)** The IRF6 expression and IRF6 promoter methylation in 372 stomach cancer samples of TCGA were analyzed by using LinkedOmics web server.
