# Supplementary figures and images for: IRF6 Is Directly Regulated by ZEB1 and ELF3, and Predicts a Favorable Prognosis in Gastric Cancer
Source: Front Oncol. 2019 Apr 4;9:220. doi: 10.3389/fonc.2019.00220 (PMC6458252; doi:10.3389/fonc.2019.00220)

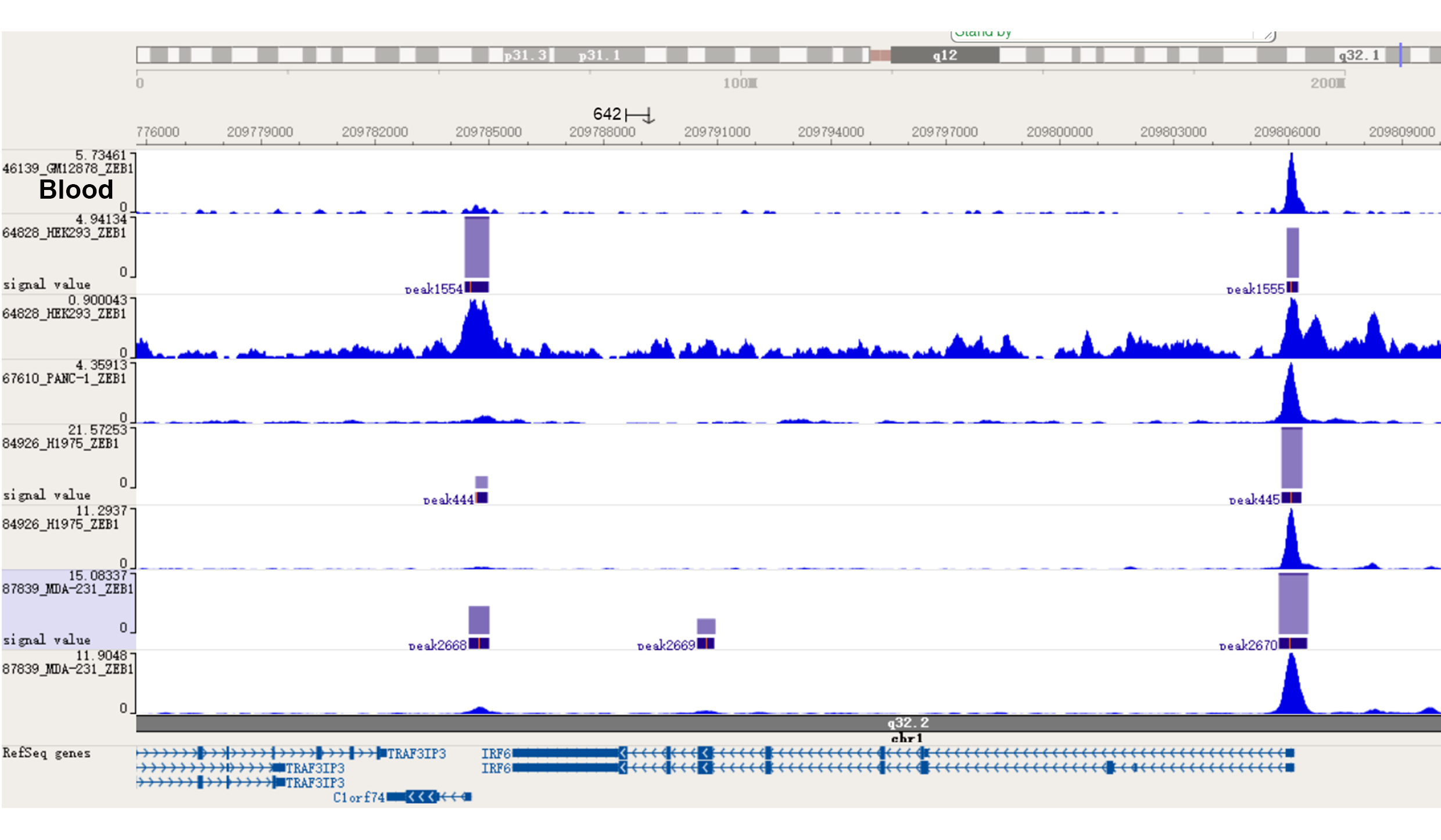

Supplement: Figure S1 — The chip-seq data of ZEB1 in different cancer cell lines was analyzed by Cistrome web server. An obvious peak located in the promoter region of IRF6 was observed in blood, kidney, pancreas, lung, and breast tissues. [file Image_1.TIF]

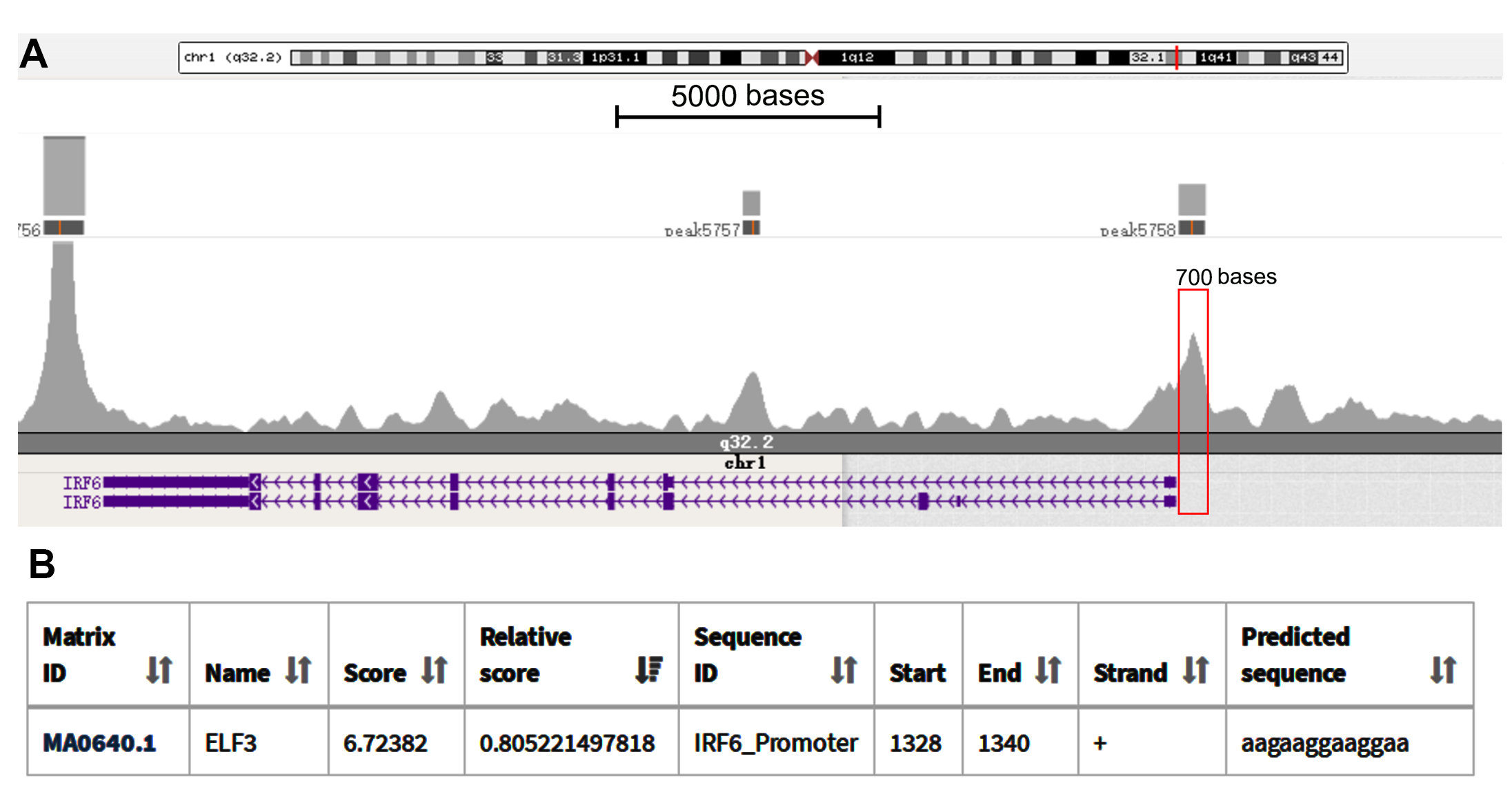

Supplement: Figure S2 — Bioinformatics analysis showed that ELF3 might be a transcriptional regulator of IRF6. (A) The chip-seq data of ELF3 in pancreatic cancer cell lines was analyzed by Cistrome web server. An obvious peak located in the 700 bp-length of promoter region of IRF6 was observed (B) The possible ELF3 binding sites in 2,000 bp-length of IRF6 promoter was analyzed by JASPAR. [file Image_2.TIF]

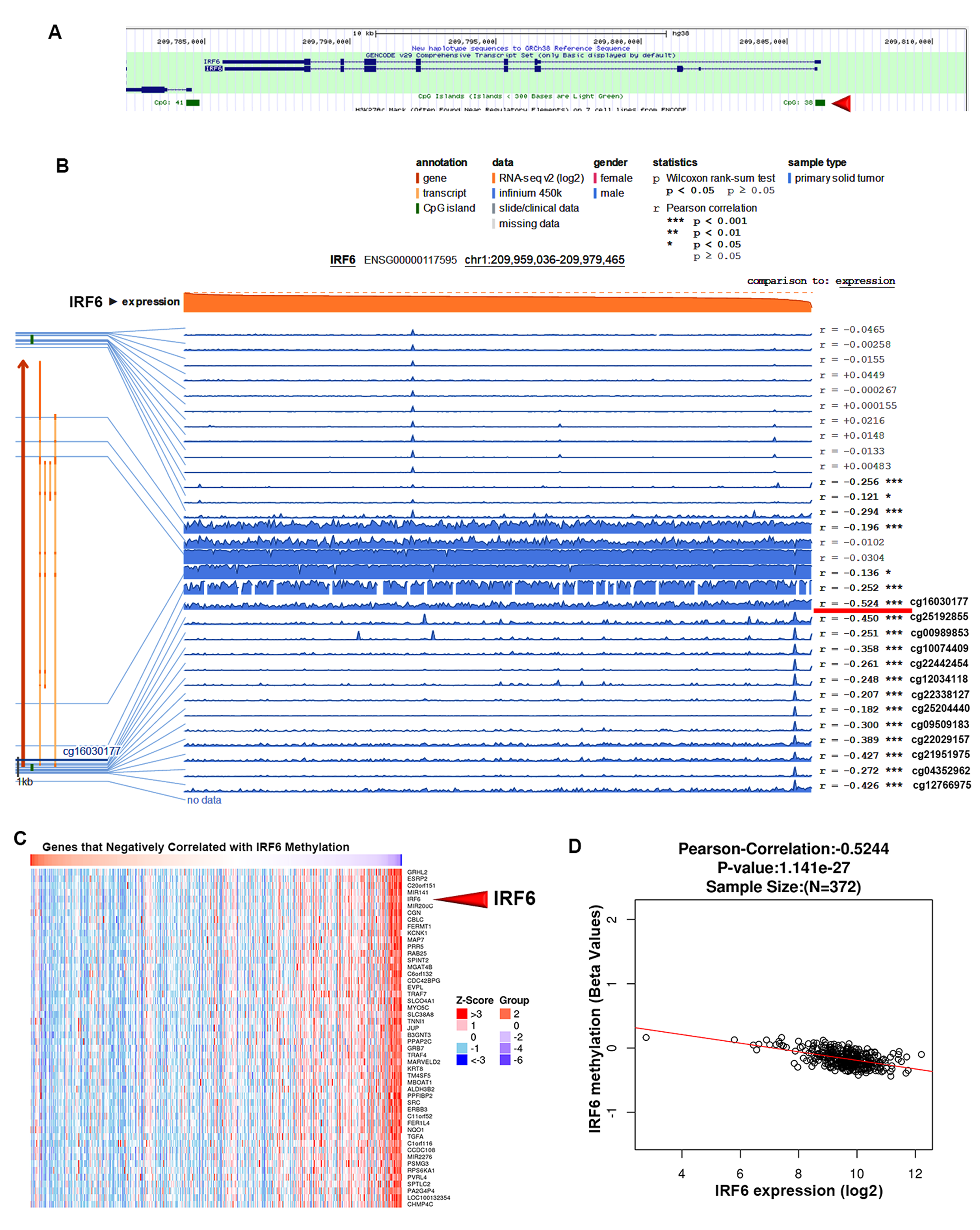

Supplement: Figure S3 — The IRF6 expression was negatively related to IRF6 promoter methylation. (A) An obvious CpG islands was located in the promoter region of IRF6. (B) The association between IRF6 expression and IRF6 promoter methylation in the TCGA stomach cancer cohort was analyzed by MEXPRESS web tool. (C) Genes that negatively correlated with IRF6 methylation was analyzed by using LinkedOmics web server. (D) The IRF6 expression and IRF6 promoter methylation in 372 stomach cancer samples of TCGA were analyzed by using LinkedOmics web server. [file Image_3.TIF]
